# Supplementary material for: Empirical methods for controlling false positives and estimating confidence in ChIP-Seq peaks
Source: BMC Bioinformatics. 2008 Dec 5;9:523. doi: 10.1186/1471-2105-9-523 (PMC2628906; doi:10.1186/1471-2105-9-523)
Supplement: Additional File 1 — A variety of html documents from the USeq web site detailing the available applications, their best usage, output file type descriptions, command line menus, etc. [file 1471-2105-9-523-S1.zip › USeqUserGuides/JavaDoc.zip!/JavaDoc/cgar/beans/LoginBean.html]

LoginBean


|  |  |  |
| --- | --- | --- |
| **Overview** \| **Package** \| **Class** \| **Use** \| **Tree** \| **Deprecated** \| **Index** \| **Help** | |  |
| **PREV CLASS**   **NEXT CLASS** | **FRAMES**    **NO FRAMES**     **All Classes** |  |
| SUMMARY: NESTED \| FIELD \| CONSTR \| METHOD | DETAIL: FIELD \| CONSTR \| METHOD |  |


---


## cgar.beans Class LoginBean

```
java.lang.Object
  cgar.beans.LoginBean
```

---

``` public class LoginBean extends java.lang.Object ```

---

**Constructor Summary**

`LoginBean()`

`LoginBean(java.lang.String loginName)`


| **Method Summary** | |
| --- | --- |
| `boolean` | `checkParams(SQL sqlConnection)` |
| `java.lang.String` | `getLoginName()` |
| `java.lang.String` | `getMessages()` |
| `java.lang.String` | `getPassword()` |
| `int` | `getUserId()` |
| `boolean` | `paramsPresent()`             Look for params. |
| `void` | `setLoginName(java.lang.String string)` |
| `void` | `setMessages(java.lang.String string)` |
| `void` | `setPassword(java.lang.String string)` |
| `boolean` | `updateSucessfulLogin(SQL sql, java.lang.String IP)`             Updates the User and Login tables to reflect a successful login. |

**Methods inherited from class java.lang.Object**

`equals, getClass, hashCode, notify, notifyAll, toString, wait, wait, wait`

**Constructor Detail**

### LoginBean

```
public LoginBean()
```

---


### LoginBean

```
public LoginBean(java.lang.String loginName)
```


**Method Detail**

### paramsPresent

```
public boolean paramsPresent()
```

:   Look for params. Set labgroup to none if empty.

---


### checkParams

```
public boolean checkParams(SQL sqlConnection)
```

---


### updateSucessfulLogin

```
public boolean updateSucessfulLogin(SQL sql,
                                    java.lang.String IP)
```

:   Updates the User and Login tables to reflect a successful login.

---


### getLoginName

```
public java.lang.String getLoginName()
```

---


### getPassword

```
public java.lang.String getPassword()
```

---


### setLoginName

```
public void setLoginName(java.lang.String string)
```

---


### setPassword

```
public void setPassword(java.lang.String string)
```

---


### getMessages

```
public java.lang.String getMessages()
```

---


### setMessages

```
public void setMessages(java.lang.String string)
```

---


### getUserId

```
public int getUserId()
```


---


|  |  |  |
| --- | --- | --- |
| **Overview** \| **Package** \| **Class** \| **Use** \| **Tree** \| **Deprecated** \| **Index** \| **Help** | |  |
| **PREV CLASS**   **NEXT CLASS** | **FRAMES**    **NO FRAMES**     **All Classes** |  |
| SUMMARY: NESTED \| FIELD \| CONSTR \| METHOD | DETAIL: FIELD \| CONSTR \| METHOD |  |


---
